# P06-03 Associations between walkability and physical activity of Hungarian adults, preliminary study of the EUPASMOS project

**DOI:** 10.1093/eurpub/ckac095.088

**Published:** 2022-08-29

**Authors:** Alexandra Makai, Viktória Prémusz, Tamás Dóczi, Réka Veress, Paolo Rocha, Pongrác Ács

**Affiliations:** 1 Faculty of Health Sciences, University of Pécs, Pécs, Hungary; 2 Department of Social Sciences, University of Physical Education, Budapest, Hungary; 3 Hungarian Leisure Sport Association, Hungarian Leisure Sport Association, Budapest, Hungary; 4 Faculty of Human Kinetics, University of Lisbon, Lisbon, Hungary

**Keywords:** physical activity, walking, cycling, accelerometer, walkability

## Abstract

**Background:**

Walkability is a new definition in sport and health science in Hungary. In our study the walkability was measured by NEWS-Hungarian questionnaire first time in Hungary and our research aim was to compare neighbourhood walkability to physical activity level.

**Methods:**

The study comprised 593 participating adults (248 males and 345 females). The data was collected during February-May 2019 using quota sampling by age and gender representing the Hungarian adult (18+) population. The physical activity was measured by RM 42 hip-worn triaxial accelerometer for 7 consecutive days using vigorous, moderate to vigorous activities and daily steps scores. The walkability was measured by walking and cycling facilities subscale (10 questions) of NEWS-Hungarian questionnaire. The subscale measured the environment's possibilities and quality for walking and cycling. Data were presented as mean ± standard deviation, Spearman's rank correlation was used to analyse data using SPSS 24. program, where level of significance was set at p > 0.05.

**Results:**

The mean age of the participants was 44.41±18.64 and their average number of daily steps was 7308.47±6993.86. 69.05% was lived in cities and 30.05% in rural areas. The respondent's opinion about the walking and cycling around their place of living was measured by a 4 point scale (1 = strongly disagree and 4 = strongly agree), where we found 2.76±2.8 mean scores. The walkability score was showed significant but weak correlation with accelerometer-measured vigorous PA (R = 0.124, p = 0.004). But the accelerometer-based number of steps was not showed correlation with walkability (R = 0.058, p = 0.184).

**Conclusions:**

According to our results the built environment, especially walking and cycling places had significant but weak effect on physical activity patters. The participants were somewhat satisfied with walking and cycling possibilities of their built environment but this was not affected directly the time spent physically active. This study used first time the NEWS-Hungarian walkability scale to have a better understanding of the country specific details and compared them with PA level of the population further analysis needed.

